# The Problem of Syphilis in Poor-Law Infirmaries

**Published:** 1914-05-02

**Authors:** 


### The Hospital

**The Workers' Newspaper of Administrative Medicine and Institutional Life, Administration, National Insurance and Health.**

No. 1453, Vol. LVI. SATURDAY, MAY 2, 1914. PRICE ONE PENNY.

### THE PROBLEM OF SYPHILIS IN POOR-LAW INFIRMARIES.

At the thirty-second meeting of the Royal Commission on Venereal Diseases, as we briefly note elsewhere, evidence was given by Dr. C. Thackray Parsons, the medical superintendent of Fulham Infirmary, on behalf of the Infirmary Medical Superintendents' Society, as to the extent to which modern methods in the treatment and diagnosis of syphilis are in use in the Metropolitan and provincial Poor-Law infirmaries. It was important that this evidence should be given, because the information hitherto available is incomplete and misleading. Dr. Johnstone, for instance, in his Report on Venereal Diseases made to the Local Government Board in July 1913, says: "Salvarsan treatment for syphilis has been given on a small scale in one or two infirmaries, and occasionally Wassermann tests are undertaken." Again, Sir Malcolm Morris, when pleading in June 1913 for the appointment of a Royal Commission to Inquire into Venereal Diseases, after referring to the very meagre provision made at the general hospitals, added: "To say that no better state of things prevails in the Metropolitan Poor-Law infirmaries is probably an understatement. I am not in possession of detailed information as to these institutions, but in three in which inquiries were made it was found that no use whatever is made of salvarsan, owing, no doubt, to the cost." The statistics collected by Dr. Parsons have not yet been published, but from inquiries we have made ourselves it would appear that salvarsan, or neo-salvarsan, is more or less systematically used in fourteen out of twenty-five London infirmaries and in thirteen out of eighteen provincial ones. The Wassermann test is employed in a somewhat smaller proportion of the infirmaries—the test being carried out in practically all cases, not at the infirmary, but in private laboratories. At the Fulham Infirmary, we believe, treatment by salvarsan has been used since April 1911—that is, from the time salvarsan was first placed on the market. More recently neo-salvarsan has been used, and Dr. Parsons notes, as do most observers, that it is less efficacious but is better tolerated than the older drug. Again, the evidence is practically unanimous that both drugs are most efficacious in acquired syphilis, less so in congenital syphilis, and attended by only temporary benefit in the case of parasyphilitic conditions.

To say that salvarsan has revolutionised the treatment of syphilis is to state what is now a generally accepted fact, but the effect of this revolution upon the institutional treatment of the disease is not so generally realised. In pre-salvarsan days it was generally acknowledged that the patient needed a mercurial course extending over at least two years and was in an infectious condition so long as there were any primary or secondary lesions in existence—a condition which often lasted for many months. The difficulty which existed in inducing patients to remain in a Poor-Law infirmary during this long period of infectivity was known to every Poor-Law medical officer. The possibility of meeting this difficulty by obtaining powers of compulsory detention was often discussed, but it was obvious that compulsion would only raise fresh difficulties and would prevent many syphilitic patients from applying for treatment in Poor-Law institutions. By the use of salvarsan, however, the problem is greatly simplified. There is a fairly general agreement among syphilologists that a course of combined mercurial and salvarsan injections extending over a period of three months is more efficient than a two years' course of mercury alone. Not only that, but the spirochætes may disappear from accessible lesions within a few hours after an intravenous injection of salvarsan, and the lesions themselves heal much more rapidly than under a course of mercurial treatment. From a public health point of view it would appear to be necessary only to detain the patient in an institution until the lesions have healed—a matter of three or four weeks in most cases—and then to arrange for him to attend weekly in order to receive an injection of mercury or salvarsan until he has completed a three months' course.

There can be no doubt that salvarsan ought to be used in all institutions undertaking the treatment of syphilis. The fact that many Poor-Law infirmaries have not yet adopted it, and that many are without special lock wards, suggests that in London and the larger provincial towns it might be desirable to specialise some of the infirmaries for the treatment of venereal diseases, and to arrange that all the cases should be sent to these infirmaries. At the meeting referred to, attention was also drawn to the lack of provision for pathological work in the infirmaries. The provision of a central laboratory, in which the Wassermann test and the examination of material for the presence of the *spirochæta pallida* and the gonococcus could be carried out, was advocated. The staff of most Poor-Law infirmaries seldom includes a skilled pathologist, and the expense of having these investigations carried out in private laboratories materially reduces the extent to which they can be used. In fact, the provision of a central pathological laboratory for Poor-Law infirmaries, not only in connection with venereal diseases, but also in the routine investigation of all forms of disease, is one of the most urgent reforms required in our Poor-Law system. The Local Government Board possesses a laboratory and pathologists employed in carrying out research work. It would not be difficult to extend this provision, so as to meet the pathological requirements of the Poor-Law infirmaries, and no more useful reform could engage the attention of the new President, Mr. Herbert Samuel.

